# Zebrafish imaging reveals hidden oncogenic–normal cell communication during primary tumorigenesis

**DOI:** 10.1247/csf.23026

**Published:** 2023-05-11

**Authors:** Yukinari Haraoka, Mai Miyake, Tohru Ishitani

**Affiliations:** 1 Department of Homeostatic Regulation, Research Institute for Microbial Diseases, Osaka University, Suita, Osaka 565-0871, Japan; 2 Center for Infectious Disease Education and Research (CiDER), Osaka University, Suita, Osaka 565-0871, Japan

**Keywords:** zebrafish, imaging, cell-cell communication, cell competition, EDAC, senescence, primary tumorigenesis

## Abstract

Oncogenic mutations drive tumorigenesis, and single cells with oncogenic mutations act as the tumor seeds that gradually evolve into fully transformed tumors. However, oncogenic cell behavior and communication with neighboring cells during primary tumorigenesis remain poorly understood. We used the zebrafish, a small vertebrate model suitable for in vivo cell biology, to address these issues. We describe the cooperative and competitive communication between oncogenic cells and neighboring cells, as revealed by our recent zebrafish imaging studies. Newly generated oncogenic cells are actively eliminated by neighboring cells in healthy epithelia, whereas oncogenic cells cooperate with their neighbors to prime tumorigenesis in unhealthy epithelia via additional mutations or inflammation. In addition, we discuss the potential of zebrafish in vivo imaging to determine the initial steps of human tumorigenesis.

## Introduction

As the proverb “seeing is believing” goes, direct observation of unknown things deepens our knowledge. For instance, the British scientist Robert Hooke discovered a “cell” through optical observation more than 350 years ago. Hooke observed sections of cork with a compound microscope, describing the smallest unit that constitutes a living organism as a “cell” (Hooke, 1665). Classical optical imaging analyses have paved the way for studying cellular biology, and modern imaging analyses have contributed to extraordinary discoveries.

Specifically, in vivo imaging analysis of zebrafish is highly effective in investigating spatiotemporal cellular behavior and communication in living vertebrates. Zebrafish are an excellent animal model for in vivo imaging because of their characteristics, such as transparency, small body size, and genetic similarities to humans (Ali *et al.*, 2011). Therefore, zebrafish have been used in cell and developmental biology and disease studies. In this review, we introduce our recent zebrafish imaging studies that revealed the hidden communication between oncogenic and neighboring cells during primary tumorigenesis and discuss the potential of zebrafish in cancer cell biology.

## Conventional approaches are insufficient to study the primary tumorigenesis mechanisms

Tumor cells harbor multiple somatic mutations. Previous studies using cultured cells, animal models, and samples from patients with cancer have shown that genetic mutations play considerable roles in tumor development. Several genes have been identified as oncogenes or tumor suppressors that promote or prevent normal cell transformation into cancer cells. The artificial introduction of oncogenes (e.g., Ras) into normal cultured cells led to their transformation into cancerous cells (Land *et al.*, 1983). In addition, by analyzing human colorectal cancer specimens, frequently mutated tumor-suppressor genes, including *TP53* and *APC*, were identified (Baker *et al.*, 1989; Kinzler *et al.*, 1991; Nishisho *et al.*, 1991).

Moreover, the pathological changes in human colorectal cancer are associated with the ordered accumulation of somatic mutations in specific oncogenes and tumor-suppressor genes, known as “Vogelgram” (Fearon and Vogelstein, 1990). These studies suggest that most cancers arise from a single oncogenic cell with a mutation in oncogenes or tumor-suppressor genes and that oncogenic cells acquire additional mutations to evolve into tumors. Furthermore, recent cancer studies using advanced cancer animal models (e.g., mouse and zebrafish cancer models induced by chemicals and genetic modifications) and next-generation sequencing have contributed substantially to understanding the mechanisms of cancer development and malignant transformation (Frese and Tuveson, 2007; Caldas, 2012; Pandey *et al.*, 2013; White *et al.*, 2013; McCreery and Balmain, 2017).

Although these studies have accelerated cancer research progression, they have primarily focused on the later stages of tumorigenesis, when cancerous lesions are already identifiable. Moreover, the mechanisms underlying the early stages of tumor development remain largely unclear. In particular, the intrinsic behavior of newly generated oncogenic cells and their communication with surrounding normal cells during primary tumorigenesis remain unexplored. Therefore, a prospective approach using animal models is necessary to clarify the initial tumorigenic process in living organisms. Specifically, an animal model for direct observation of tumor formation from a single oncogenic cell is essential.

Nevertheless, oncogenic mutations were introduced into whole organs rather than single cells in most previous studies using animal models, such as mice. In addition, performing live-imaging analysis to observe tumorigenesis in mice is extremely challenging because of their low transparency and large body sizes. A longer tumor development period is also a disadvantage in mouse models. Thus, to elucidate the primary tumorigenesis process, we need an animal model to directly and continuously observe the behavior of a small number of oncogenic cells and their surrounding normal cells in living tissues during tumorigenesis. Zebrafish is a suitable animal model to fulfill this requirement since zebrafish larvae are transparent and small, enabling us to easily capture oncogenic cell behavior using microscopy. Considering the advantages of using zebrafish, we performed in vivo imaging to visualize the behavior of single oncogenic cells and their communication with neighboring cells.

## An unexpected competitive communication between oncogenic and neighboring cells revealed by zebrafish imaging

**Fig. 1:**
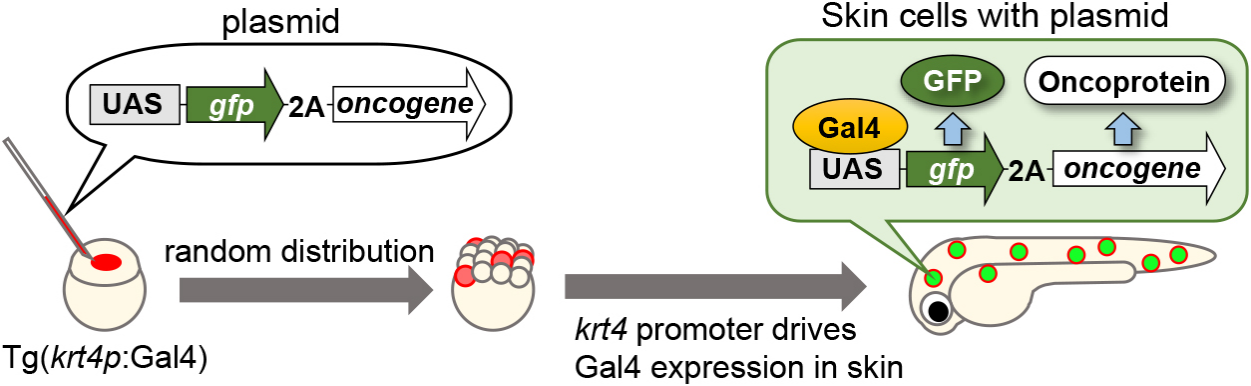
Schematic illustration of the mosaic introduction of oncogenic cells on zebrafish larval skin Abbreviations: UAS, upstream activation sequence; 2A, 2A self-cleaving peptides; *krt4*, *keratin4*.

As most tumors arise from epithelial tissues, we used the epidermal skin of zebrafish larvae as a model for human epithelial tissues. Zebrafish larval skin is structurally similar to human epithelial tissue and has been proposed as a valuable model of human epithelial tissue (Eisenhoffer *et al.*, 2017). Therefore, we developed a method to visualize how oncogenic cells prime tumorigenesis in the zebrafish larval skin using the Gal4-UAS system (Fig. 1) (Akieda *et al.*, 2019; Takeuchi *et al.*, 2020; Haraoka *et al.*, 2022). By injecting upstream activation sequence (UAS) promoter-driven oncogenic protein expression plasmids, fluorescent protein (e.g., GFP or mCherry)-expressing oncogenic cells were artificially introduced into the skin of *keratin4* (*krt4*)-*gal4*-transgenic zebrafish larvae. Cells with UAS-driven plasmids were randomly distributed in the larval body, and the fusion proteins (fluorescent protein-2A-oncoprotein) were expressed and then cleaved at the self-cleaving 2A peptide sequence (2A), leading to the expression of fluorescent proteins and oncoproteins in the larval skin, where the *krt4* promoter drove Gal4 expression. Consequently, fluorescent oncogenic cells appeared sporadically on the larval skin (Fig. 1).

**Fig. 2:**
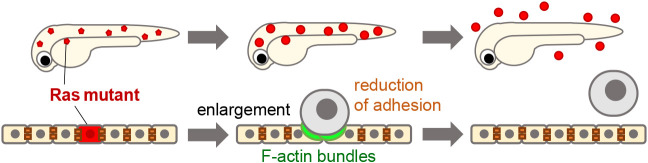
Schematic illustration of the mechanisms of mosaic Ras mutant cell elimination from zebrafish skin

Using this system, we introduced oncogenic cells harboring the oncogenic Ras^G12V^ mutation. Ras mutations are observed in ~30% of human cancers, and the Ras^G12V^ mutation is the most common oncogenic Ras mutation found in tumors (Bos, 1988; Prior *et al.*, 2012). First, we expected that mosaic cells with the Ras^G12V^ mutation would form primary tumors on the skin. Unexpectedly, the Ras mutant cells gradually swelled and were apically extruded from the larval skin (Fig. 2). Mechanistically, in response to the appearance of Ras mutant cells, neighboring cells induced cellular senescence and irreversible cell cycle arrest. Subsequently, senescence triggered cell enlargement and reduction in adhesion to neighboring cells, which drove the “physical elimination” of Ras mutant cells from the epithelia. The surrounding normal cells formed F-actin bundles, generating a driving force for mutant cell elimination (Haraoka *et al.*, 2022). Thus, our zebrafish imaging analyses revealed previously unknown antitumor mechanisms.

A similar antitumor mechanism has also been reported in mammalian cells. Mosaically introduced Ras mutant cells were apically extruded through communication with neighboring normal cells in Madin–Darby canine kidney cell cultures and mouse small intestine, pancreas, and lungs (Hogan *et al.*, 2009; Kon *et al.*, 2017; Sasaki *et al.*, 2018). Similar to our observations in zebrafish, neighboring normal cells formed F-actin bundles (Hogan *et al.*, 2009) in these mammalian cells. Although the molecular mechanisms of this oncogenic cell elimination, known as an epithelial defense against cancer or cell competition, have been well studied (Kajita and Fujita, 2015; Kon and Fujita, 2021), the involvement of senescence has not been reported. We believe that using zebrafish to investigate in vivo molecular mechanisms enables the discovery of new mechanisms. Most researchers have explored molecular and cellular mechanisms using mammalian cell cultures and have confirmed that they work in vivo using mouse models. However, it is challenging to determine detailed in vivo mechanisms using mammalian models.

In contrast, zebrafish can be used to examine these mechanisms in vivo. However, mammals are better human models than zebrafish. Therefore, collaboration with mammalian research may facilitate understanding primary tumorigenesis mechanisms. In collaboration with Dr. Fujita, we identified two calcium signaling modes, calcium waves and calcium sparks, as new evolutionarily conserved mediators of oncogenic cell elimination. In response to the appearance of oncogenic Ras mutant cells, mechanosensitive calcium channel-mediated transient upsurges in intracellular calcium ions, called calcium sparks, frequently occur in neighboring normal cells. Subsequently, calcium sparks stimulate the movement of normal and Ras mutant cells, promoting apical mutant cell extrusion (Takeuchi *et al.*, 2020; Kuromiya *et al.*, 2022). In addition to the calcium spark, just before the apical extrusion, a wavy release of calcium ion (calcium wave) occurred from Ras mutant cells to surrounding normal cells. This calcium influx led to the apical elimination of Ras mutant cells by increasing the polarized movement of surrounding cells toward extruding Ras mutant cells (Kuromiya *et al.*, 2022; Takeuchi *et al.*, 2020). Furthermore, these calcium signals are required for physical cellular movement and neighboring cell-mediated Ras mutant cell senescence (Haraoka *et al.*, 2022). Thus, combined analyses using zebrafish and mammalian models effectively clarify evolutionarily conserved primary tumorigenesis mechanisms.

## Novel anti-tumorigenic role and regulation of cellular senescence

**Fig. 3:**
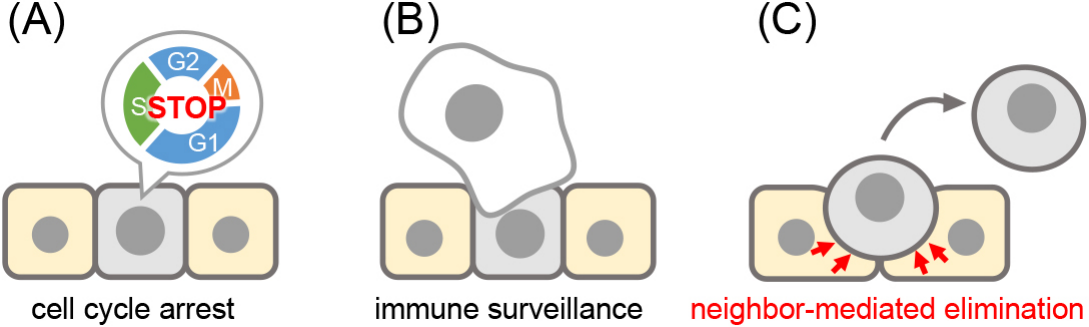
Schematic illustration of three anti-tumorigenic roles of cellular senescence (A) The induction of irreversible cell cycle arrest in oncogenic cells, (B) The stimulation of immune cell-mediated senescent cell elimination, and (C) Neighboring normal cell-mediated elimination of oncogenic cells.

Previous studies using mammalian models have shown that cellular senescence suppresses tumorigenesis through two mechanisms (Collado and Serrano, 2010; Gorgoulis *et al.*, 2019): the induction of permanent cell cycle arrest in oncogenic cells (Fig. 3A) and immune surveillance triggering to facilitate oncogenic cell elimination (Fig. 3B). In addition, our zebrafish imaging analyses clarified that neighboring normal cell-mediated elimination of oncogenic cells is the third tumor-suppressive mechanism of cellular senescence (Haraoka *et al.*, 2022) (Fig. 3C).

**Fig. 4:**
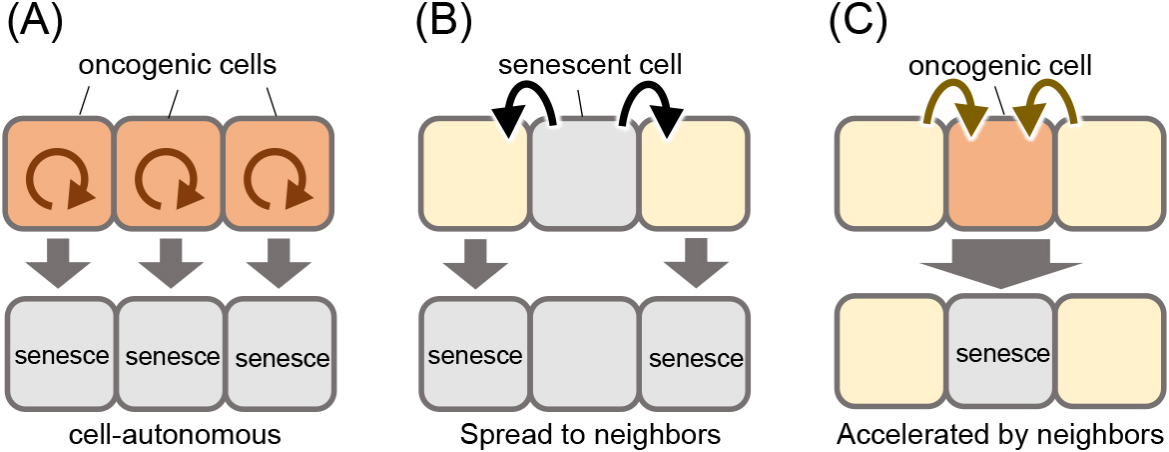
Schematic illustration of three mechanisms of OIS (A) Oncogenic signaling-induced cellular senescence in a cell-autonomous manner. (B) Senesced oncogenic cells spread senescence to neighboring cells through cell-cell communication. (C) Intercellular communication with neighboring normal cells accelerated senescence in oncogenic signaling-activated cells.

The mechanisms of oncogene-induced cellular senescence (OIS) have been extensively studied in mammalian cell cultures, as follows: oncogenic signaling, such as hyperactive Ras signaling, stimulates cellular senescence in a cell-autonomous manner (Serrano *et al.*, 1997) (Fig. 4A), and senesced oncogenic cells spread cellular senescence to neighboring cells through cell-cell communication (Acosta *et al.*, 2013) (Fig. 4B). Our imaging analyses revealed that in vivo OIS was accelerated by intercellular communication with neighboring normal cells in zebrafish skin (Haraoka *et al.*, 2022) (Fig. 4C). Ras mutant cells require several days to senesce after Ras activation in cultured cells (Serrano *et al.*, 1997). In contrast, in zebrafish skin, Ras hyperactivated mutant cells surrounded by normal cells undergo senescence within several hours (Haraoka *et al.*, 2022). We also demonstrated that neighboring normal cells are required for senescence induction (Haraoka *et al.*, 2022). Our findings suggest that sporadically generated oncogenic cells immediately senesce after communicating with normal cells in living tissues. Thus, in vivo imaging has the potential to clarify mechanisms that cannot be detected in cell culture.

## Additional mutation prevents oncogenic cell elimination to prime tumorigenesis

**Fig. 5:**
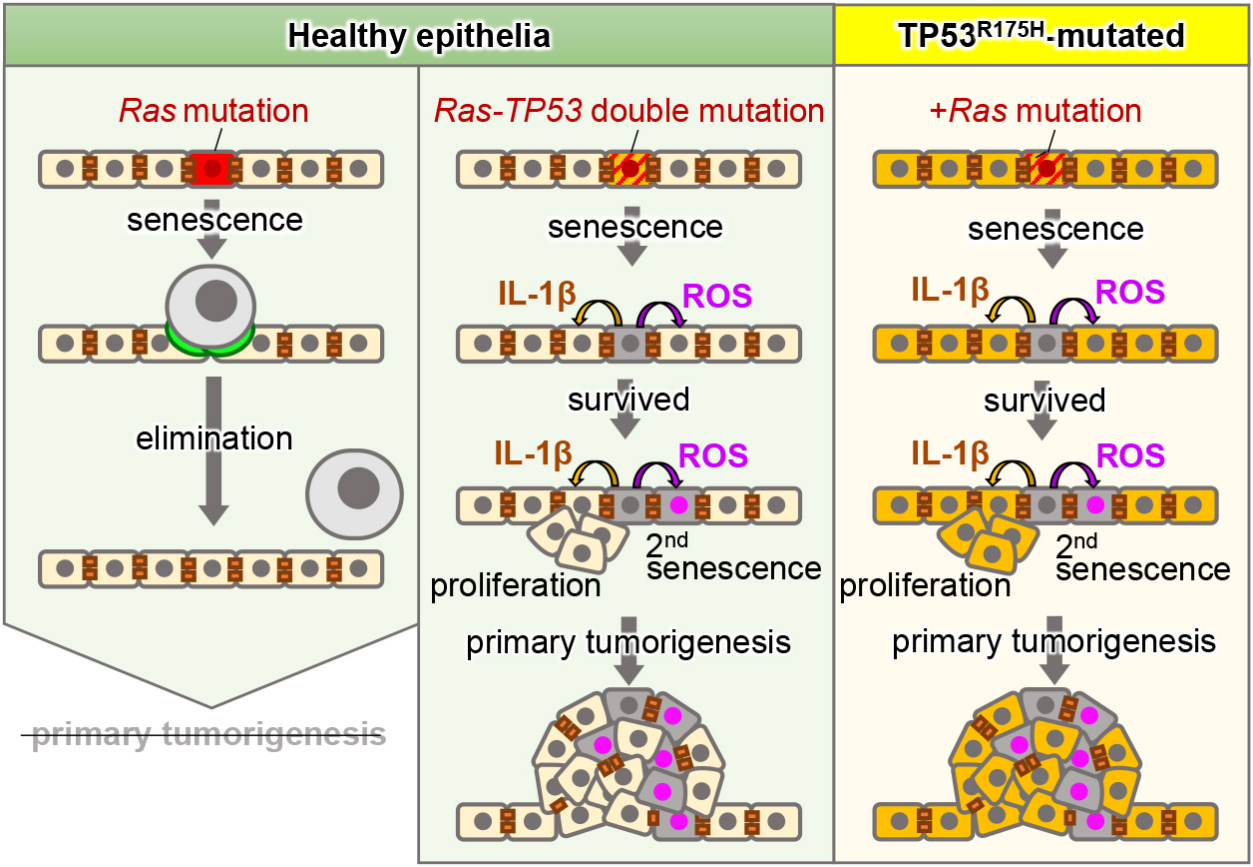
Schematic illustration of the behaviors of Ras mutant and Ras-TP53 double mutant cells and their neighboring cells on healthy and unhealthy epithelia

Although animals possess a system that eliminates newly generated oncogenic cells, it is unclear why tumors are generated. Based on cancer genome studies, somatic mutation accumulation is hypothesized to be the fundamental cause of tumorigenesis (Stratton *et al.*, 2009; Vogelstein *et al.*, 2013; Garraway and Lander, 2013). However, the significance of this mutation accumulation has not yet been demonstrated. Therefore, we examined mutation accumulation using our zebrafish imaging system and found that additional mutations in the tumor-suppressor gene *TP53* prevented this elimination and primed tumorigenesis. Specifically, the introduction of TP53R175H, TP53R248W, or TP53R273H, all of which are *TP53* gain-of-function hotspot mutations (Muller and Vousden, 2014), into Ras mutant cells blocked F-actin accumulation in neighboring cells and membrane E-cadherin reduction, and consequently the cells survived in zebrafish skin (Fig. 5, middle). Survived Ras-TP53 double mutant cells also underwent cellular senescence and secreted inflammatory factors, including interleukin (IL)-1β, IL-6, IL-8, and reactive oxygen species (ROS), which are called senescence-associated secretory phenotype (SASP) factors (Coppé *et al.*, 2010). Double mutant cell-secreted IL-1β stimulated neighboring cell proliferation, whereas ROS derived from double mutant cells induced cellular senescence in neighboring cells. Subsequently, secondary senescent cells secreted SASP factors facilitating neighboring cell proliferation and senescence, forming heterogeneous primary tumors (Haraoka *et al.*, 2022). Thus, oncogenic cells with Ras-TP53 double mutations in healthy epithelia can prime tumorigenesis by evading neighboring cell-mediated elimination (Fig. 5, middle).

However, how TP53-mutated unhealthy epithelia respond to the Ras mutation introduction is still unclear. We have previously found that Ras mutation-introduced cells in epithelia with TP53 gain-of-function mutations (Ras-TP53 double mutant cells on TP53-mutated epithelia) survived and formed primary tumors via a mechanism similar to that of double mutant cell-mediated primary tumorigenesis in healthy epithelia (Fig. 5, right).

Notably, TP53 loss-of-function did not prevent oncogenic cell elimination or prime tumor formation in zebrafish skin, indicating that gain-of-function mutations, but not loss-of-function mutations, in TP53 switch the fate of oncogenic cells from death to survival. Consistent with these findings, mutations in both the K-Ras and TP53 genes are frequently observed in various human cancers (Fearon and Vogelstein, 1990; Maitra and Hruban, 2008), and published cancer genomics dataset (publicly available at http://www.cbioportal.org) indicates that K-Ras and TP53 mutations co-occurred in approximately 60% of human pancreatic adenocarcinoma cases and in about 25% of human colorectal adenocarcinoma cases. Furthermore, TP53 gain-of-function mutations are frequently detected as hotspot point mutations in human cancers and enhance oncogenic potential (Muller and Vousden, 2014; Olive *et al.*, 2004; Lang *et al.*, 2004). Thus, the mechanisms observed in our zebrafish model may be similar to those observed in humans.

## The potential common mechanisms driving the initial process of tumorigenesis

Epithelial tissues can eliminate a variety of oncogenic cells. Similar to Ras mutant cells, oncogenic cells expressing the Rous sarcoma virus Src gene (v-Src), which was the first identified oncogene (Hunter and Sefton, 1980), are apically extruded from mammalian epithelial sheets and zebrafish embryonic and larval epithelia when they are surrounded by normal cells (Kajita *et al.*, 2010; Anton *et al.*, 2018; Haraoka *et al.*, 2022).

**Fig. 6:**
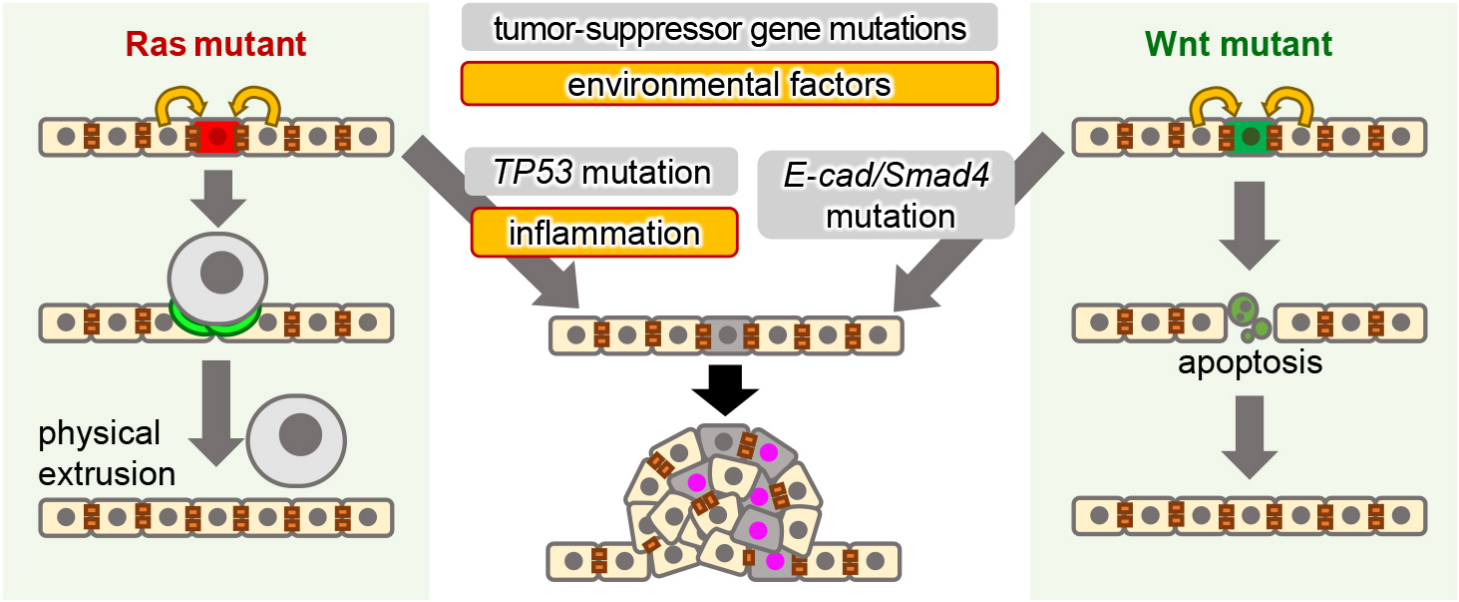
Schematic illustration of the fate of Ras or Wnt mutant cells and the effects of additional mutations in tumor-suppressor genes and inflammation

Recently, we also found that oncogenic cells with abnormal Wnt/β-catenin signaling activity are eliminated through communication with normal cells (Akieda *et al.*, 2019). Mutations in Wnt/β-catenin signaling components are detected in 80% of colorectal carcinomas (Schepers and Clevers, 2012; White *et al.*, 2012), 30–40% of hepatocellular carcinomas (de La Coste *et al.*, 1998; Miyoshi *et al.*, 1998; Satoh *et al.*, 2000), and 80–90% of hepatoblastomas (Cairo *et al.*, 2008; Armengol *et al.*, 2011). Zebrafish imaging analyses revealed that oncogenic cells with abnormally high Wnt/β-catenin signaling activity were apoptotically eliminated through communication with neighboring normal cells (Akieda *et al.*, 2019). Wnt/β-catenin signaling-hyperactivated cells increased the membrane levels of E-cadherin protein, leading to substantial differences in membrane E-cadherin levels (cadherin-imbalance) between unfit and neighboring normal cells. Neighboring cells sense Wnt/β-catenin-hyperactivated cells through cadherin-imbalance and stimulate the nuclear translocation of transcription factors Smad2/3/4, leading to ROS production, anti-apoptotic protein Bcl-2 degradation, and consequent apoptosis induction in Wnt/β-catenin-hyperactivated cells (Akieda *et al.*, 2019) (Fig. 6 Right). Notably, E-cadherin and Smad4, the essential mediators of this elimination system, are also tumor-suppressor genes (Christofori and Semb, 1999; Derynck *et al.*, 2001), and loss-of-function of either E-cadherin or Smad4 prevented Wnt/β-catenin-hyperactivated cells from apoptotic elimination in zebrafish (Akieda *et al.*, 2019). This relationship between Wnt/β-catenin signaling (oncogenic signaling) and E-cadherin/Smad4 (tumor-suppressor genes) is very similar to that between Ras signaling and TP53. Although the elimination mode is distinct between oncogenic signaling types, the neighboring normal cell-mediated oncogenic cell elimination with aberrant oncogenic signaling and its disruption by tumor-suppressor gene mutations may involve common mechanisms driving the initial tumorigenesis step (Fig. 6). Recent studies using mouse models and mammalian cell cultures have also shown that tumor-suppressor protein loss reduces oncogenic cell elimination activity (Kohashi *et al.*, 2021), which might be evolutionarily conserved from fish to humans.

## Environmental factors abrogate oncogenic cell elimination and lead to tumorigenesis

Tumorigenesis progress through intrinsic (e.g., somatic mutations) and extrinsic factors (e.g., environmental stress) (Wu *et al.*, 2018). Similar to Ras-TP53 double mutant cells on healthy epithelia, Ras mutant cells evade elimination and form heterogeneous tumors on unhealthy epithelia with inflammation or DNA damage (Fig. 6, left). Inflammatory cytokine IL-1β expression was sufficient to reduce Ras mutant cell-induced F-actin formation in neighboring cells, thereby inhibiting Ras mutant cell elimination. DNA damage enhanced IL-1β expression and ROS production; IL-1β blocks mutant cell elimination and neighboring cell proliferation, and ROS senesces neighboring cells, resulting in heterogeneous tumor formation. Agreeing with our findings, obesity-induced inflammation and hyperinsulinemia also prevented oncogenic cell elimination, leading to cancer in mouse and fly models (Sasaki *et al.*, 2018; Sanaki *et al.*, 2020). Thus, environmental factors appear to play crucial roles in controlling the oncogenic cell behavior and priming tumorigenesis.

## A new mode of heterogenous tumor formation discovered by zebrafish imaging

One of the features of Ras-TP53 double mutant cell-induced tumors is heterogeneity; that is, they contain mutant and neighboring cells. Tumors are consistently described as heterogeneous cell populations, known as “intra-tumor heterogeneity.” Intra-tumor heterogeneity is the differences in molecular signatures between tumor cells within a single tumor and can induce therapy resistance (Dagogo-Jack and Shaw, 2018). Understanding the causes of intra-tumoral heterogeneity is crucial to overcoming this therapeutic resistance. According to a well-accepted hypothesis, differentiation, additional mutations, or both, in cancer cells are thought to induce tumor heterogeneity (Dexter and Leith, 1986; Beck and Blanpain, 2013). In contrast, our zebrafish study suggests a new, different model: that original cancerous cells convert neighboring cells to abnormal proliferative or senescent cells through SASP factor (ROS and IL-1β)-mediated communication, thereby forming a heterogeneous cell mass (Fig. 5). Because ROS can induce genomic mutations (Waris and Ahsan, 2006), ROS-exposed neighboring cells would acquire new genetic mutations, also contributing to the heterogeneity induction. Thus, we successfully unraveled new potential mechanisms underlying primary tumorigenesis using zebrafish imaging analyses.

## Conclusion and perspective

Numerous molecules and mechanisms regulating cancer development have been discovered in mammalian cell cultures, mouse models, and human patient samples. However, the mechanisms underlying primary tumorigenesis remain unclear due to technical limitations. Our recent studies using zebrafish in vivo imaging uncovered the hidden molecular and cellular mechanisms underlying the ultra-early stage of tumorigenesis. Future studies using zebrafish imaging will facilitate understanding the entire picture of primary tumorigenesis in living animals.

As the final topic of this review, we identify the unclear questions raised in our studies and our new goal. The first question concerns how newly emerging oncogenic cells are sensed and identified as cells that should be eliminated. Although recent cell competition studies have identified a few molecules for sensing (e.g., PTP10D, LILR3B, and E-cadherin) (Yamamoto *et al.*, 2017; Ayukawa *et al.*, 2021; Akieda *et al.*, 2019), the sensing mechanisms have not yet been well studied. Moreover, it is still unclear whether a common sensing logic applies to various oncogenic cells. To comprehensively understand the communication mechanisms between oncogenic cells and their neighbors during primary tumorigenesis, in vivo imaging and new cutting-edge technologies are required. Although we recently developed a transcriptomic method for oncogenic cells in zebrafish using fluorescence-activated cell sorting (FACS) and succeeded in identifying the detailed mechanisms in oncogenic cells using this method (Akieda *et al.*, 2019), we could not obtain the transcriptome of neighboring cells because tissues should be dissociated before FACS. To clarify the sensing mechanisms and to eliminate oncogenic cells, the transcriptomes of neighboring cells would be helpful. Therefore, we are applying a recent unique transcriptomics technology, photo-isolation chemistry, enabling us to obtain a high-depth transcriptome specifically from photo-irradiated regions of interest (Honda *et al.*, 2021) to examine the cell-cell communication mechanisms during primary tumorigenesis in zebrafish. The zebrafish imaging and spatial omics combination will facilitate the identification of new regulators that specifically function in the ultra-early stages of tumorigenesis. We could use these specific regulators to develop a novel technology for the early detection and prevention of cancer.

We have succeeded in “synthesizing” a primary tumor in zebrafish by introducing oncogenic mutations with tumor-suppressor gene mutations or environmental factors. These findings suggest that adding tumor-suppressor gene mutations or environmental factors is required to prime tumorigenesis. An “in vivo synthetic approach” would facilitate the identification of essential events for tumorigenesis, which are challenging to clarify using conventional approaches, including mouse models and cell cultures. Notably, the Ras-TP53 double mutant cell-induced primary tumor in zebrafish seemed benign and did not invade or metastasize to other tissues. Therefore, we are now searching for additional stimulations (e.g., additional mutations or environmental stresses) that can induce the malignant transformation of this benign tumor in zebrafish. We aim to identify the new factors necessary for tumor malignancy using this approach.

A new advantage of the zebrafish model is that phenotype-based drug screening has attracted the attention of medical and pharmaceutical researchers. Because zebrafish larvae are microscopic and kept in minimal volumes of water, we tested the effects of drugs on zebrafish phenotypes by treating them with very small amounts of drugs and typical multi-well plastic plates, as with drug screening using mammalian cell cultures. Several drugs that control hematopoietic stem cell proliferation or treat hearing loss have been isolated from zebrafish drug screens (North *et al.*, 2007; Coffin *et al.*, 2010). Notably, our zebrafish system can generate primary tumors within several hours of oncogenic cell appearance (Haraoka *et al.*, 2022), whereas mouse models require several weeks for tumor generation (Guerin *et al.*, 2020). This indicates that the short-term effects of anti-cancer drug candidates can be evaluated. Considering these and other advantages, this zebrafish system is an excellent model for rapidly screening anti-cancer drug candidates.

We believe that zebrafish in vivo imaging sheds light on hidden cellular communications and contributes to understanding the mechanisms underlying primary tumorigenesis and developing novel anti-cancer approaches.
